# Social Acceptance of Renewable Energy Technologies in the Post-fukushima Era

**DOI:** 10.3389/fpsyg.2020.612090

**Published:** 2021-01-15

**Authors:** Eunil Park

**Affiliations:** Sungkyunkwan University, Seoul, South Korea

**Keywords:** Fukushima, renewable energy, nuclear energy, social acceptance, attitude

## Abstract

In 2011, the Fukushima nuclear accident occurred, and this had a strong effect on public perceptions of energy facilities and services that relate not only to nuclear energy, but also renewable energy resources. Moreover, the accident has also considerably affected national energy plans in both developing and developed countries. In South Korea, several studies have been conducted since the accident to investigate public perspectives toward particular energy technologies; however, few studies have investigated public perceptions of renewable-energy technologies and tracked the transitions. Therefore, this study examines the trend of South Korean public’s perceptions of renewable-energy technologies. Based on data collected in 2016, we validated the structural connections and determined that trust, benefits, risks, and attitude were key determinants of the public’s desire to adopt these technologies; specifically, public attitude was found to be the greatest determinant of this desire. Based on the results, both implications and limitations are examined.

## Introduction

In March 2011, the Fukushima nuclear power plant was struck by a huge tsunami caused by a 9.0-magnitude earthquake approximately 180 km east of Japan’s Tohoku region. This seriously damaged the plant, resulting in three meltdowns. The nuclear accident had serious negative effects on the regional and global environment; moreover, the accident also led to a fundamental paradigm shift in most nations in regard to their energy policies (Wittneben, 2012).

The Fukushima nuclear accident also affected public perspectives toward nuclear-energy technologies and facilities; however, several studies have shown that there are notable differences in such public perceptions across different nations (Visschers and Siegrist, 2013; Bird et al., 2014; Richter et al., 2015). Furthermore, compared to the breadth of prior research on public perceptions of nuclear-energy technologies and facilities, few studies have focused on public perceptions of technologies and facilities relating to alternative energy (Cherp and Jewell, 2016; Komiyama and Fujii, 2017).

Public perception of alternative-energy sources is considered one of the most important factors influencing the investment allocated to related energy facilities and technologies within national energy plans. Moreover, these perceptions are also affected by events and accidents in other countries (Gamson and Modigliani, 1989; Verplanken, 1989). After the Fukushima accident caused significant public resistance toward nuclear energy, the majority of both developed and developing countries that were considering using nuclear energy as their main energy and electricity supply resource have completely reviewed and revised their national energy plans (Dhakal, 2009; Chen et al., 2014). For instance, the German government has changed its national energy policies to exclude nuclear energy from its future energy plans and the Japanese government has revised its national energy plan to exclude nuclear energy as a primary energy resource (Betzer et al., 2013; Hong et al., 2013).

The Fukushima nuclear accident allowed the public in most countries to learn of the serious negative effects of nuclear energy technologies and facilities on global environments and citizens’ health (Shimura et al., 2015). Consequently, this created a public desire for the implementation of alternative energy resources in order to reduce the usage of nuclear energy. Amongst the various energy resources that are currently under consideration to replace nuclear energy, renewable-energy resources are considered to be one of the most promising (Mbarek et al., 2015).

As renewable-energy resources may play an important role in revised future energy plans, several studies have explored public perceptions of renewable energy. For example, Bang et al. (2000) found that consumer concerns toward renewable energy were notable determinants of consumer attitude toward willingness to adopt renewable energy. Painuly (2001) also indicated that there are various barriers to employing renewable energy in developing countries. In addition, the research of Mallett (2007), and Wüstenhagen and Boehnke (2008) have shown that the public’s economic, socio-demographic, and psychological factors can form significant determinants of the public’s desire to adopt renewable-energy technologies. However, few studies have focused on the transitions of public attitudes toward renewable-energy technologies (Park and Ohm, 2014).

In South Korea, Park and Ohm (2014) examined public perceptions of renewable energy technologies, proposed an adoption model for renewable energy technologies, and conducted pen-and-paper surveys both before and after the Fukushima nuclear accident. Before the accident, cost was one of the main reasons behind the inhibited usage of renewable-energy technologies; however, after the accident, public attitudes toward the technologies and their perceived low-degree of risk became notable determinants toward desire to adopt the technologies (Park and Ohm, 2014).

Consequently, the current study attempts to explore the following points:

1. Has there been any change in public perceptions of renewable-energy technologies since the Fukushima nuclear accident?
2. What has motivated the public to adopt renewable-energy technologies in South Korea?

In order to address the first research question, the current study reviews the findings and results of Park and Ohm (2014), conducts a pen-and-paper survey in South Korea, and tracks the notable changes in public perspectives toward renewable-energy technologies. As mentioned by Park and Ohm (2014), because there is a substitutional relationship between renewable and nuclear energy it would be worthwhile to present the effects the Fukushima nuclear accident had on public perceptions toward renewable-energy technologies and examine significant changes in the public perceptions in South Korea in this regard.

Considering the second point, the current study uses the acceptance model for renewable-energy technologies tested by Park and Ohm (2014). Based on the results of the structural-equation modeling method, we can determine the motivations behind the public adoption of the technologies, and then compare the results of the current study with those of prior studies.

The remainder of this study is organized as follows: after presenting the findings of prior studies that have focused on the adoption of renewable energy, the study methodology is examined. The results and key findings are then presented. Finally, the limitations and future studies are examined.

## Literature Review and Hypotheses

### Social Acceptance of Renewable Energy

In order to respond both global warming and environmental pollutions, several nations significantly focus on both facilities and policies of alternative energy resources (Gielen et al., 2019). It means that utilizing alternative energy resources and employing a mixed energy plans are one of the important tasks in establishing the national energy policies (Dagoumas and Koltsaklis, 2019). Moreover, there have been notable efforts in using renewable energy resources for both national and local energy plans (Young and Brans, 2020).

However, there are significant economic, social, and industrial encumbrances related to utilizing renewable energy resources in the plans (Cajot et al., 2017). Among them, social perceptions of specific energy resources and technologies are considered as one of the principal issues in the regional and national levels (Paravantis et al., 2018). With no careful comprehensive procedures on specific energy-related facilities, a number of local or national conflicts can be presented (Kwon, 2018). Because of this reason, a number of researchers and public officials have investigated how to explore social perceptions of specific energy resources, including renewable energy resources (Kim et al., 2020).

Ribeiro et al. (2014) conducted a survey of public opinions on four renewable energy technologies, solar, hydro, biomass, and wind power. Considering 3,646 respondents, they found that there are a positive public perspective toward renewable energy resources, whereas NIMBY syndrome is significantly presented in the areas with biomass facilities. Moreover, they indicated that utilizing solar energy resources and employing hydropower are the appropriate desirable solutions for the economic and environmental contributions, and welfare aspects, respectively.

Liu et al. (2013) attempted to address social diffusion of renewable energy technologies in one of the rural areas in China through a field survey. Based on an analytical framework developed by the theory of planned behavior, they found that rural residents tend to have supportive perspectives toward renewable energy deployment with consideration of its positive relationships with environment. The results of 212 validated responses also reported that there are notable social and economic factors in determining rural residents’ willing to pay for green electricity.

Bertsch et al. (2016) addressed public acceptance of renewable energy and its-related policy. Conducting a survey in Germany, both the national and local levels’ determinants of adopting renewable energy sources were examined. The results of a multivariate analysis of covariance showed that there were significant differences between local and national acceptance levels, while socio-demographic information (e.g., age and education) was crucially related to the acceptance levels.

Although there are a number of prior studies on social acceptance of renewable energy resources (Kim et al., 2020), there are certain obstacles to track constant changes of social perceptions and acceptance of the resources. Because time-suitable grasping social opinions is one of the important issues (Kardooni et al., 2018), presenting both potentiality and significance of consistent tracking social opinions should be presented for governmental officers and stakeholders.

### Reviews on the Acceptance of Renewable Energy Facilities in Korea

Notable quantitative studies have explored public perceptions of specific energy technologies and facilities from social-science perspectives. For instance, McGowan and Sauter (2005) showed that, in regard to national energy plans, UK citizens preferred investment in renewable-energy facilities over nuclear-energy facilities.

Moreover, although several significant studies have investigated public attitudes and the adoption of renewable-energy technologies in regional and national perspectives, a limited number of studies have explored public attitudes and energy preferences before and after nuclear accidents, which may have notable effects on the attitudes toward and adoption of particular energy technologies as well as alternative technologies (Eiser et al., 1989). One of the most notable transitions in public attitudes and adoption occurred in the 1970s when the global oil crisis caused citizens to become concerned about their national energy plans, policies, and economy. Returning to the present, in South Korea, Park and Ohm (2014) proposed an integrated research model for adopting renewable-energy technologies, and captured the significant transitions in public attitudes between the periods before and after the Fukushima accident. Considering seven factors, the main determinants of public desire to adopt renewable-energy technologies changed from cost to attitude.

**FIGURE 1:**
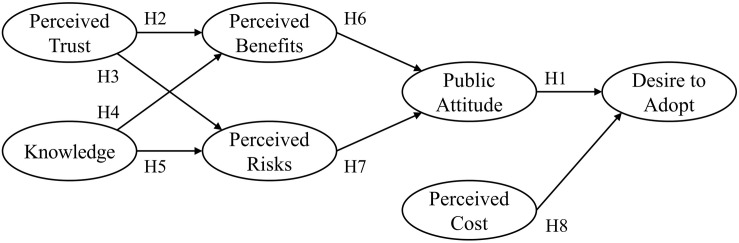
The proposed research model used in this study.

For investigating the transitions, surveying citizens’ opinions is considered one of the most accurate and successful research approaches. Consequently, the current study employs the conceptual research model previously validated by Park and Ohm (2014), and captures the notable changes in public perceptions of renewable-energy technologies over time. In the research model of Park and Ohm (2014), the following hypotheses are considered (Figure 1):

H1. A higher degree of attitude leads to a higher degree of desire to adopt.

H2. A higher degree of perceived trust leads to a higher degree of perceived benefits.

H3. A higher degree of perceived trust leads to a lower degree of perceived risks.

H4. A higher degree of knowledge leads to a higher degree of perceived benefits.

H5. A higher degree of knowledge leads to a lower degree of perceived risks.

H6. A higher degree of perceived benefits leads to a higher degree of public attitude.

H7. A higher degree of perceived risks leads to a lower degree of public attitude.

H8. A higher degree of perceived cost leads to a lower degree of desire to adopt.

## Study Method

### Survey Design and Procedure

Following the procedures of the survey-design methodology used by Park and Ohm (2014), the current study employed identical questionnaire items to those used in the main survey of Park and Ohm (2014).

We followed all procedures presented by Park and Ohm (2014): (1) exploring unique characteristics, (2) presenting selected constructs, (3) examining the potentiality and validity of the constructs, (4) conducting a pilot test with validity tests, and (5) presenting the survey.

In addition to two-time survey sessions in 2010 and 2012, which were conducted in prior studies (Park and Ohm, 2014), we conducted additional survey in 2016. All questionnaire items, sampling procedures and outliner filtering methodologies (a stratified quota sampling) were identical with prior survey sessions in 2010 and 2012. The survey was distributed to 1,500 potential respondents in 6 regions and 18 cities in South Korea. In order to ensure the representativeness of the sample in the survey, the current study applied a stratified quota-sampling method. After excluding incomplete and invalidated responses, 991 (66.1% of response rate) samples were used in the statistical analysis.

### Measurements

All measurements in this study were validated by prior studies (Park and Ohm, 2014). All participants were instructed to mark each item with a 7-point Likert scale (7: strongly agree/1: strongly disagree). The perceived trust was examined by three items (Cronbach’s alpha: 0.890; e.g., “*I believe that renewable energy technologies can improve our energy generation industry successfully.*”). Three items contributed to the perceived benefits (Cronbach’s alpha: 0.912; e.g., “*Renewable energy technologies may help us develop increased industrial competitive advantages.*”). Moreover, the perceived cost was presented by three items (Cronbach’s alpha: 0.888; e.g., “*I think the maintenance cost of using renewable energy technologies and generators is expensive*”), while three items composed the perceived risks (Cronbach’s alpha: 0.921; e.g., “*Renewable energy technologies and plants can harm our society including animals and plants.*”). Three items were employed to examine the desire to adopt (Cronbach’s alpha: 0.909; e.g., “*If I could, I would prefer to use renewable energy technologies and generators.*”). The public attitude was presented by three items (Cronbach’s alpha: 0.879; e.g., “*Applying renewable energy technologies is extremely good for us*”). Lastly, the public knowledge was organized by three items (Cronbach’s alpha: 0.904; e.g., “*how familiar are you with renewable energy sources and technologies?*”).

## Data Analysis

A structural-equation modeling (SEM) method was used to capture the structural changes in the research model. In addition, by computing the total effects of the factors relating to approval and comparing the results of the computations and SEM (2010, 2012, and 2016), the current study aims to track significant changes in the structural relationships within the research model.

## Results

### Analysis Methods

The connections in the research model were examined and analyzed using SEM. In order to test the reliability of the employed constructs, we employed confirmatory-factor analysis. The current study meets the recommendations of previous SEM studies in regard to internal (all Cronbach’s alphas were higher than 0.7), convergent (all factor loadings, composite reliability, and average variance extracted values were higher than 0.7, 0.7, and 0.5, respectively), and discriminant reliability tests (The correlation values between two specific constructs were lower than the square rots of the average variance extracted).

### Fit Indices

The current study computed the fit indices of the measurement and research models by considering if the collected data were well-represented by the measurement and research models. The fit indices of the measurement and research models were found to be acceptable (Table 1).

**TABLE 1:** The fit indices of the measurement and research models (M: The measurement model, R: The research model; Anderson and Gerbing, 1988; Bagozzi and Yi, 1988; Jöreskog and Sörbom, 1996; Kenny and McCoach, 2003; Hoe, 2008).

Fit indices	Before the Fukushima accident	Post-Fukushima		Satisfaction levels
	2010	2012	2016	
χ^2^/d.f.	4.08*^*M*^*, 4.07*^*R*^*	4.44*^*M*^*, 4.01*^*R*^*	4.32*^*M*^*, 4.32*^*R*^*	<5.00
Normed fit index	0.94*^*M*^*, 0.95*^*R*^*	0.91*^*M*^*, 0.93*^*R*^*	0.90*^*M*^*, 0.90*^*R*^*	>0.80
Incremental fit index	0.97*^*M*^*, 0.96*^*R*^*	0.94*^*M*^*, 0.91*^*R*^*	0.92*^*M*^*, 0.92*^*R*^*	>0.90
Comparative fit index	0.94*^*M*^*, 0.94*^*R*^*	0.92*^*M*^*, 0.90*^*R*^*	0.92*^*M*^*, 0.91*^*R*^*	>0.80
Goodness-of-fit index	0.95*^*M*^*, 0.94*^*R*^*	0.96*^*M*^*, 0.92*^*R*^*	0.93*^*M*^*, 0.90*^*R*^*	>0.80
Adjusted goodness-of-fit index	0.95*^*M*^*, 0.95*^*R*^*	0.95*^*M*^*, 0.94*^*R*^*	0.93*^*M*^*, 0.92*^*R*^*	>0.80
Standardized root mean square residual	0.05*^*M*^*, 0.06*^*R*^*	0.05*^*M*^*, 0.05*^*R*^*	0.07*^*M*^*, 0.07*^*R*^*	<0.08
Root mean square error of approximation	0.04*^*M*^*, 0.05*^*R*^*	0.05*^*M*^*, 0.05*^*R*^*	0.06*^*M*^*, 0.07*^*R*^*	<0.08

### Hypothesis Testing

#### Structural Results of the Research Model

**FIGURE 2:**
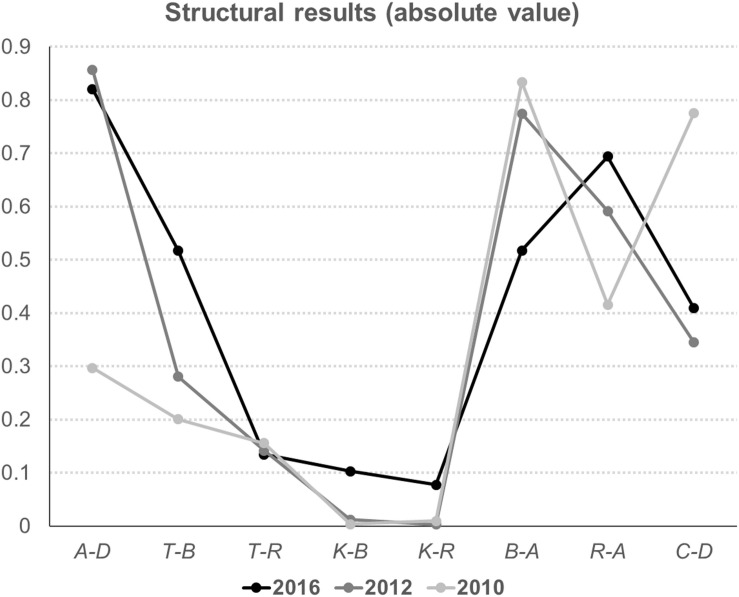
Summary of the results from 2010, 2012, and 2016 (A > Attitude; D > Public desire to adopt; T > Perceived trust; B > Perceived benefits; R > Perceived risks; K > Public knowledge; C > Perceived cost).

The structural results of the research model are summarized in Table 2, and a comparison is presented in Figure 2. The results of the data that was collected in 2016 supported six hypotheses, while two hypotheses concerning knowledge-benefits and knowledge-risks were not significant (H4, β = −0.103, *CR* = −4.789, *p* > 0.05; H5, β = −0.078, *CR* = −2.822, *p* > 0.5). Public desire to adopt renewable technologies was significantly determined by two factors, public attitude and perceived cost, while the effects of public attitude on the desire to adopt (H1, β = 0.821, *CR* = 81.712, *p* < 0.001) were greater than those of perceived cost (H8, β = −0.409, *CR* = −45.766, *p* < 0.001). Perceived benefits had positive effects on the attitude (H6, β = 0.518, *CR* = 72.988, *p* < 0.001), while the attitude was negatively affected by perceived risks (H7, β = −0.694, *CR* = −79.218, *p* < 0.001).

**TABLE 2:** Summary of the structural results from 2016 (*p < 0.001).

Hypothesis	Standardized path coefficient	SE	CR	Results
H1. Attitude → Adoption	0.821*	0.038	81.712	Supported
H2. Trust → Benefits	0.517*	0.041	68.105	Supported
H3. Trust → Risks	−0.135*	0.029	–5.877	Supported
H4. Knowledge → Benefits	–0.103	0.037	–4.789	Not supported
H5. Knowledge → Risks	–0.078	0.045	–2.822	Not supported
H6. Benefits → Attitude	0.518*	0.044	72.988	Supported
H7. Risks → Attitude	−0.694*	0.025	–79.218	Supported
H8. Cost → Adoption	−0.409*	0.031	–45.766	Supported

**Attitude > Public attitude; Adoption > Public desire to adopt; Trust > Perceived trust; Benefits > Perceived benefits; Risks > Perceived risks; Knowledge > Public knowledge; Cost > Perceived cost.**

#### Sum of Total Absolute Effects on the Intention

**FIGURE 3:**
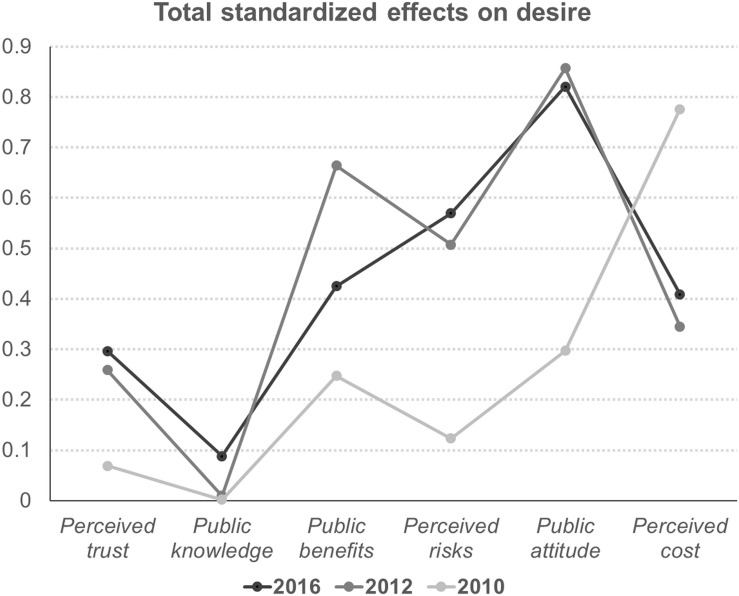
Summary of the effects on desire to adopt.

In order to present the key motivations behind users’ attitudes toward renewable-energy technologies, the total standardized effects of motivations and barriers in regard to this attitude were computed. Table 3 and Figure 3 present a summary of the total effects on desire to adopt. Although the effect perceived cost had on desire to adopt significantly diminished between 2010 and 2012 (0.776 → 0.345), it became moderately influential in 2016 (0.409). Compared to the role of cost, public attitude consistently remained a main determinant of desire to adopt (0.821 in 2016). The effects of perceived risks of adoption are becoming more important (0.124 in 2010 → 0.507 in 2012 → 0.570 in 2016), while the effects of perceived benefits of adoption are abating (0.248 → 0.664 → 0.425). Although public knowledge of renewable-energy technologies has been increasing, the effects of public knowledge are still lower than those of perceived trust (0.088 and 0.297).

**TABLE 3:** Total standardized effects on the desire to adopt.

Year	Trust	Knowledge	Benefits	Risks	Attitude	Cost
2016	0.297	0.088	0.425	0.570	0.821	0.409
2012	0.259	0.009	0.664	0.507	0.857	0.345
2010	0.069	0.002	0.248	0.124	0.297	0.776

**Trust > Perceived trust; Knowledge > Public knowledge; Benefits > Perceived benefits; Risks > Perceived risks; Attitude > Public attitude; Cost > Perceived cost.**

#### Sum of Total Absolute Effects on the Attitude

**FIGURE 4:**
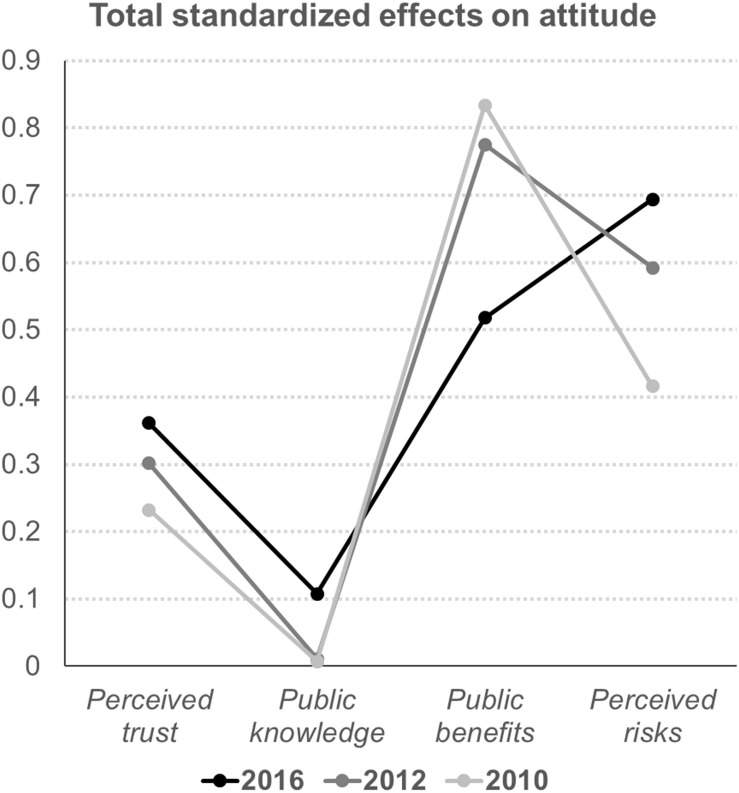
Summary of the effects on attitude.

The transitions of the effects of perceived trust, public knowledge, public benefits, and perceived risks on public desire to adopt renewable-energy technologies were quite similar to those concerning attitude. In addition, public attitude played a notable role in affecting desire to adopt, while the effects of perceived cost on desire to adopt have increased. Similar to the effects of the constructs on adoption, the roles of public knowledge, perceived trust, and risks in regard to determining public attitude have also been growing in importance (Table 4 and Figure 4). However, the effects of perceived benefits on attitude have reduced (0.775 → 0.518).

**TABLE 4:** Total standardized effects on attitude.

Year	Trust	Knowledge	Benefits	Risks
2016	0.362	0.107	0.518	0.694
2012	0.302	0.011	0.775	0.592
2010	0.233	0.007	0.834	0.416

**Trust > Perceived trust; Knowledge > Public knowledge; Benefits > Perceived benefits; Risks > Perceived risks.**

## Conclusion

This study aims to track users’ perceptions of renewable-energy technologies in the “post-Fukushima era.” Based on the findings of a previously conducted study on the South Korean public’s perception of such technologies, this study re-examines the research model used in this previous study and investigates the effects of the employed antecedents on public attitude toward and desire to adopt renewable-energy technologies. This study aimed to track the effects the Fukushima nuclear accident had on public perspectives toward renewable-energy technologies in South Korea. As previous studies, both before and after the incident, have fragmentarily observed public perceptions on such technologies, this study conducted a survey in 2016, 5 years after the accident. Then, we compared the results of the data collected by the survey and the results of previous studies. Two factors, the perceived risks and benefits, significantly affected the attitude, while the risks and benefits were mainly determined by perceived trust in the technologies. Although two factors, the risks and benefits, which were confirmed in prior research as determinants of public desire to adopt such technologies, are also presented in this study as the antecedents of the desire to adopt, there are notable transitions in the post-Fukushima era (Park and Ohm, 2014).

Since the Fukushima nuclear accident, the South Korea public has tended to adopt more risk-oriented perspectives toward particular energy technologies. It means that H7 was magnified after the Fukushima accident in South Korea [Total standardized effects (TSE): 0.416 (2010) → 0.592 (2012) → 0.694 (2016)]. In regard to motivations, a more comprehensive understanding of perceived trust is developing (H2 and H3); moreover, public knowledge of renewable-energy technologies is becoming important in forming public attitude toward and desire to adopt the technologies [H4 and H5; TSE: 0.007 (2010) → 0.011 (2012) → 0.107 (2016)].

This means that citizens are becoming familiar with renewable-energy technologies, and are beginning to understand the potential risks and benefits of such technologies (H7). Although the Fukushima nuclear accident, which occurred in a country close to South Korea, was not directly associated with renewable-energy technologies, the results of the current study provide notable evidence that the incident has continually and consistently influenced the public’s perceptions of particular energy technologies.

Moreover, the results from 2016 also contribute to providing a better understanding of the sequential relationships of users’ perceived trust-benefits and risks, attitude, and desire to adopt, and also show the significant roles perceived risk and trust in renewable-energy technologies play in regard to the diffusion, distribution, and success of the technologies in South Korea.

## Implications, Limitations, and Future Studies

Consistent with the findings of prior studies, the current study validates the structural connections between desire to adopt, attitude, benefits (and risks), and trust in renewable-energy technologies in South Korea. However, some transitions were observed in 2016 that conflicted with the results from 2010 and 2012. Two variables, public attitude and perceived cost, still affect public desire to adopt renewable-energy technologies; however, perceived risk is becoming more important. Although public benefits is still significant, its significance in determining public attitude has become more moderate compared to its status in 2010 and 2012.

Although the Fukushima accident does not have direct connections with renewable-energy technologies, it has led to notable lessons for the public. After the accident, the potential risks of energy technologies, which are mainly dependent on perceived trust, are beginning to become one of the most significant determinants of public attitude. Moreover, the key determinant of public attitude has changed from perceived benefits to risks. This means that citizens are more concerned about the potential harmfulness of energy technologies than their advantages. Although the effects of public knowledge are slight, the importance and significance of this knowledge are increasing.

As presented in the results, trust is still the key determinant of benefits and risks. This means that the South Korean government and its industry should be more responsible and make its national energy plans sustainable and eco-friendly, focusing on the distribution of renewable-energy technologies and aiding public usage and consumption of the technologies.

In effect, the government and industry should focus on the revision of legislation, the enforcement of ordinances and regulations, the provision of subsidies and benefits, and the incubation of social trust in renewable-energy technologies. Moreover, the government should include the public as one of the key participants in the decision-making process concerning the revision, provision, and incubation of energy policies.

Although the current study presents some findings, there are several limitations. First, for several reasons, it is not easy to generalize the results of the current study. For example, because the survey described in this study was conducted in South Korea, regional and cultural characteristics may have had an effect on the public’s perceptions. Second, the current study applies a research model for public perceptions that was validated in prior research along with the motivations tested therein (Park and Ohm, 2014). Several studies have indicated that other motivations can be significantly related to the adoption of energy technologies (Assefa and Frostell, 2007; Huijts et al., 2012). Consequently, future research should address these limitations and extend the findings of the current study.

## Data Availability Statement

All datasets generated for this study are included in the article/supplementary material, further inquiries can be directed to the corresponding author/s.

## Ethics Statement

The studies involving human participants were reviewed and approved by the Department of Interaction Science, Sungkyunkwan University. The patients/participants provided their written informed consent to participate in this study.

## Author Contributions

EP fully conducted and wrote the manuscript.

## Conflict of Interest

The author declares that the research was conducted in the absence of any commercial or financial relationships that could be construed as a potential conflict of interest.
